# Formula 1 drivers demonstrate superior concentration and confidence compared to Formula 2 drivers

**DOI:** 10.3389/fpsyg.2026.1697498

**Published:** 2026-06-04

**Authors:** P. J. McKnight, R. Manwaring, L. Felton

**Affiliations:** 1 Sport Department, ProPath, Snö Performance, Abu Dhabi, United Arab Emirates; 2 Performance Department, Red Bull Racing, Milton Keynes, United Kingdom; 3 School of Psychology, Sport and Health Sciences, University of Portsmouth, Portsmouth, United Kingdom

**Keywords:** ACSI-28, concentration, elite performance, Formula 1, motorsport psychology, psychological skills, self-confidence

## Abstract

This study represents the first investigation of psychological skills in Formula 1 (F1) and Formula 2 (F2) drivers, addressing a critical gap in motorsport psychology research. Ten professional drivers (5 F1, 5 F2; all male; age = 24 ± 4 years) completed the Athletic Coping Skills Inventory-28 (ACSI-28) assessing seven psychological domains, with independent samples *t*-tests comparing groups using Cohen’s *d* effect sizes. Although total ACSI-28 scores showed no significant difference (*p* = 0.47), F1 drivers demonstrated superior concentration (M = 9.4 vs. 6.8, *d* = 1.31, *p* = 0.03) and confidence/achievement motivation (M = 11.2 vs. 9.6, *d* = 1.60, *p* = 0.04). Medium effect sizes favoured F1 drivers for coping with adversity (*d* = 0.63) and peaking under pressure (*d* = 0.56), while F2 drivers showed advantages in goal-setting (*d* = 0.34) and freedom from worry (*d* = 0.22). Elite motorsport performance is distinguished by specific psychological capabilities that relate to the demands of high-pressure competition. F1 drivers’ marked advantages in concentration and confidence suggest these domains are critical determinants of success at motorsport’s pinnacle. The pattern of F2 advantages in systematic preparation (*d* = 0.34, non-significant) alongside F1 superiority in pressure response suggests a preliminary hypothesis of developmental progression in psychological skill emphasis that warrants further investigation. These findings provide evidence-based foundations for talent development and targeted interventions in elite motorsport.

## Introduction

Open-wheel motor racing represents one of the world’s most popular and globally followed sports. The FIA Formula 1 World Championship and FIA Formula 2 Championships captivate millions of viewers worldwide through their unique combination of cutting-edge technology, extreme physical demands, and high-stakes competition. The sport’s appeal extends beyond mere entertainment, as these elite racing series showcase human performance at the absolute limits of speed, precision, and decision-making under extreme pressure. Despite this widespread popularity and the evident psychological challenges inherent in motorsport competition, there remains a striking paucity of scientific research examining the mental characteristics that distinguish elite drivers. In this regard, elite motorsport shares critical characteristics with other life-critical occupational domains, including combat aviation, air traffic control, and high-level surgery, where cognitive performance under extreme stress directly determines safety outcomes (Grassmann et al., 2017; Metzger and Parasuraman, 2006). The psychological demands of operating complex machinery at speeds exceeding 350 km/h, where split-second lapses can result in catastrophic consequences, position motorsport as a unique natural laboratory for studying human performance under conditions of maximal cognitive load and existential risk.

The lack of research in motorsport psychology represents a significant gap in our understanding of elite athletic performance. While extensive literature exists documenting psychological skills across traditional sports, motorsport has been largely overlooked by sport psychology researchers. This oversight is particularly concerning given the unique psychological demands of high-speed competition, where split-second decisions can determine not only performance outcomes but also dangerous situations. The extreme consequences of mental lapses in motorsport create performance pressures that are arguably unmatched in other sporting contexts, yet we know remarkably little about the psychological profiles that enable drivers to excel in these conditions.

### Psychological skills and coping in elite sport

In high-stakes elite sport environments, psychological skills function not merely as supportive tools but as fundamental determinants of performance, consistency, and resilience (Birrer and Morgan, 2010; Macnamara and Button, 2010). Athletes operating at the highest competitive levels must effectively manage intense pressure, sustain concentration over extended periods, regulate emotional responses, and execute split-second decisions under conditions of uncertainty and fatigue (Kent et al., 2018; Parkin et al., 2017). This psychological challenge becomes particularly acute in motorsport, where mental readiness influences not only performance outcomes but also safety considerations and career longevity.

Within high-risk sporting and occupational domains, psychological skills transcend their traditional role as performance enhancers to function as fundamental survival mechanisms. Young and Knight (2014) demonstrated that risk sport athletes employ psychological skills ‘as a form of precautionary behaviour, which prepare risk sport athletes for future adversity’ (p. 7). This framing is particularly relevant for motorsport, where attentional control and adversity coping serve dual functions: optimizing competitive performance while simultaneously maintaining the cognitive vigilance necessary to prevent potentially fatal errors. The theoretical integration of performance and safety considerations distinguishes motorsport psychology from traditional sport psychology applications and necessitates research that examines psychological skills through this dual lens.

Elite athletic performance consistently demonstrates that success in high performance sport requires continual management of constantly changing challenges (Crocker et al., 2015). Modern sport psychology recognises that optimal performance depends not solely on physical capabilities, or level of technical skills, but equally on sophisticated psychological skills that enable athletes to regulate their mental states under pressure. Psychological skills are defined as an athlete’s ability to use learned methods to regulate or enhance their psychological characteristics (Dohme et al., 2017), representing trainable competencies rather than fixed traits.

Moreover, there is evidence that psychological skills are related to several outcome variables, such as performance (Lochbaum et al., 2022). Research consistently shows that psychological skills have been known to increase a number of psychological variables (e.g., self-confidence, satisfaction, enjoyment) in athletes (Young and Knight, 2014), while also facilitating maximizing their physical skills and obtaining an enhanced sport performance (Birrer and Morgan, 2010). Elite performers demonstrate distinct psychological characteristics, with successful athletes exhibiting less depressed, less sensitive, yet more confident, more anxious and aggressive profiles compared to less successful counterparts (Bebetsos, 2003; Mahoney, 1989).

Coping represents a particularly crucial psychological skill in high-pressure environments. Lazarus and Folkman’s foundational work defines coping as involving volitional thoughts and actions to manage physically and psychologically demanding situations” (Crocker et al., 2015). In elite sport contexts, effective coping enables athletes to regulate attentional and emotional processes so to focus on task-relevant cues and keep arousal within an adaptive range (Christensen and Smith, 2016). This becomes especially critical when athletes face pressure situations characterized by the presence of incentives that result in an appraisal that the execution of a performance calls for an optimal outcome, improved performance, or enhanced functioning (Hill et al., 2011), which aptly describes the high-stakes environment of elite motorsport.

The multidimensional nature of psychological skills encompasses various domains essential for elite performance. Research indicates that successful athletes demonstrate superior abilities in mental-imaging, control of mental power, stress control, attention, concentration and goal setting (Martens, 1987; Weinberg and Gould, 2024). Concentration, defined as the power to keep the focus on the selected stimulus in a given time (Mohammadzadeh and Sami, 2014), represents a particularly crucial skill in environments requiring sustained attention amid distractions. Self-confidence, the degree of confidence in oneself ability to perform an action at a certain point of time (Mohammadzadeh and Sami, 2014), similarly distinguishes elite from non-elite performers across sporting contexts.

### Psychological skills training and development

The trainable nature of psychological skills offers significant implications for performance enhancement. Psychological skills training (PST) is a popular method which involves teaching athletes methods to help them to enhance the quality and consistency of their performance (Lange-Smith et al., 2024). Meta-analytic evidence demonstrates that PST interventions can enhance performance (Lange-Smith et al., 2024), while systematic reviews confirm significant moderate effects for psychological skills training, mindfulness-based approaches, and imagery techniques (Reinebo et al., 2024).

Importantly, psychological skills development does not occur automatically through experience alone. Research reveals that only five out of twelve mental skills showed significant development as athletes progressed from novice to experienced levels, while seven skills did not improve automatically with experience (Bochaver et al., 2021). This finding underscores the necessity for systematic psychological skills training rather than relying solely on experiential learning.

### Performance under pressure

Elite motorsport epitomises performance under extreme pressure, where an individual may be confronted with situations, where the outcome hinges on one pressured moment (Kent et al., 2018). The psychological demands extend beyond typical sporting contexts, requiring drivers to maintain split-second decisions, maintain fine motor control under physical and mental fatigue, underpinned by the knowledge that the performance outcome can result in consequences of risk or reward (Kent et al., 2018). Success in such environments depends on effective cognitive, behavioural, and emotional self-regulation skills (Crocker et al., 2015), making psychological skills assessment particularly relevant for understanding elite motorsport performance.

### Research demand and study rationale

Despite the clear importance of psychological skills in high-pressure sporting environments, motorsport psychology remains severely under-researched. To date, no published studies have specifically examined the psychological characteristics of Formula 1 and Formula 2 drivers, representing a critical gap in our understanding of elite performance in this unique sporting context. This absence of research is particularly problematic given the extreme psychological demands inherent in high-speed, high-risk competition where mental readiness influences both performance outcomes and safety considerations.

Understanding the psychological skills profile of elite motorsport competitors could provide valuable insights for talent identification protocols, driver development programs, and performance optimization strategies. Such knowledge would enable evidence-based approaches to psychological preparation in motorsport, potentially enhancing both competitive outcomes and safety standards.

Therefore, this study aims to provide the first comprehensive examination of psychological skills in Formula 1 and Formula 2 drivers using the validated Athletic Coping Skills Inventory-28 (ACSI-28), (Smith et al., 1995). Specifically, we seek to: (1) establish baseline psychological skill profiles for elite motorsport competitors, (2) identify differences in psychological characteristics between F1 and F2 drivers, and (3) provide evidence-based foundations for psychological skills training in motorsport contexts.

Based on the unique demands of elite motorsport competition and the established relationship between psychological skills and competitive level in other high-performance domains, we hypothesised that: (H1) F1 drivers would demonstrate higher overall psychological coping resources than F2 drivers, reflecting the greater psychological demands at motorsport’s pinnacle; and (H2) F1 drivers would score significantly higher on pressure-related subscales—specifically Concentration, Coping with Adversity, and Peaking Under Pressure—given the extreme attentional and emotional regulation demands inherent in F1 competition. Through addressing these objectives, this research seeks to bridge the significant gap between motorsport practice and sport psychology science, contributing essential knowledge to this understudied but critically important domain of elite athletic performance.

## Methods

### Participants

The study sample consisted of ten full-time, actively competing professional motorsport athletes, comprising five Formula 1 (F1) drivers and five Formula 2 (F2) drivers. All participants were male and engaged at the highest competitive level of their respective series during the time of data collection. Professional driving experience was operationally defined as the total number of years competing in recognised international single-seater championships, including Formula 1, Formula 2 (and its predecessor GP2), International Formula 3 (including GP3), and Formula Renault 3.5. This cumulative experience was used as a proxy for exposure to elite-level motorsport environments. Demographic information including age and years of professional driving experience was collected for each participant (Table 1), with age analysed using median split methodology and years of professional driving experience examined using a 7.5-year cutoff point.

**Table 1:** Descriptive statistics (mean ± standard deviation) for age and professional driving experience of participants by competition level.

Variable	F1 drivers (*n* = 5)	F2 drivers (*n* = 5)	All drivers (*n* = 10)
Age (years)	27 ± 4	22 ± 2	24 ± 4
Professional experience (years)	10 ± 4.1	4.6 ± 0.5	7.3 ± 3.9

### Measures

Psychological coping skills were assessed using the Athletic Coping Skills Inventory-28 (ACSI-28; Smith et al., 1995), a 28-item multidimensional self-report instrument designed to assess sport-specific psychological coping skills in athletes. The ACSI-28 comprises seven subscales, each containing four items: (1) Coping With Adversity, (2) Peaking Under Pressure, (3) Goal Setting/Mental Preparation, (4) Concentration, (5) Freedom From Worry, (6) Confidence and Achievement Motivation, and (7) Coachability.

Items are rated on a 4-point Likert scale ranging from 0 (*almost never*) to 3 (*almost always*). Six items (3, 7, 10, 12, 19, and 23) are reverse scored due to negative wording. Subscale scores are calculated as the total of the four constituent items, resulting in scores ranging from 0.0 to 12.0 for each domain. A total Personal Coping Resources score can be computed by summing all individual item responses across the seven subscales, with a maximum possible total score of 84.

### Data collection

Each driver’s individual Performance Coach was briefed by the Performance Director on the purpose of the study and introduced to the Athletic Coping Skills Inventory-28 (ACSI-28; Smith et al., 1995). To ensure ease of access and standardisation, the 28-item questionnaire was digitised and administered via Ethica Data, a mobile platform designed for health and clinical research (Avicenna Research, 2019). A link to download the app was provided to each of the 10 Performance Coaches, and the system was configured to automatically record and transmit responses to the Performance Director for central collation.

To support consistency and completion, drivers completed the questionnaire in collaboration with their Performance Coaches at the same time of year during the racing season. Coaches verbally administered the questions and recorded the drivers’ selected responses in the app without offering any interpretation or guidance. This approach ensured data accuracy and completeness, given the likelihood that drivers may not have completed the questionnaire independently.

The collaborative administration protocol, while enhancing completion rates and data accuracy, introduces potential social desirability bias that warrants acknowledgment. The presence of Performance Coaches during questionnaire completion may have influenced drivers’ responses, particularly for items assessing confidence, coping abilities, and coachability—domains where presenting a favorable self-image could be advantageous. To mitigate this risk, several procedural safeguards were implemented: (a) coaches were explicitly instructed to record responses verbatim without offering interpretation or guidance; (b) individual responses were anonymized prior to analysis and were not shared with team management or used in performance evaluations; (c) drivers were assured that their responses would have no bearing on their competitive standing or team relationships. Nevertheless, the potential for response inflation represents a methodological limitation that should be considered when interpreting results, particularly the notably high Confidence scores observed across both driver groups.

This study was approved by the Michigan State University Institutional Review Board and was conducted in accordance with ethical standards for research involving human participants and the 1964 Helsinki Declaration and its later amendments. All participants provided informed consent by clicking “I agree” to a digital consent form within the Ethica Data application, where the study purpose and procedures were explained, and participants granted permission for their data to be used anonymously for research purposes.

### Data processing

Individual item responses were processed according to standard ACSI-28 protocols with systematic reverse scoring applied to six negatively worded items (items 3, 7, 10, 12, 19, and 23). Factor scores were calculated as the total of constituent items for each psychological domain, resulting in scores ranging from 0.0 to 12.0 for each factor. A total ACSI score (i.e., Personal Coping Resources) was computed by summing all individual item responses across the seven subscales. Only participants with complete responses across all 28 items were included in the analysis.

### Statistical analysis

Descriptive statistics including means, standard deviations, and sample sizes were calculated for each psychological factor by group. Primary analyses employed independent samples *t*-tests to compare F1 and F2 drivers across all seven psychological factors and the total ACSI score. All tests utilized an alpha level of 0.05 with degrees of freedom set at 8 (*n*₁ + *n*₂ − 2) for two-tailed tests. Effect sizes were quantified using Cohen’s *d*, with interpretations following conventional guidelines where *d* = 0.2–0.5 represents a small effect, *d* = 0.5–0.8 a medium effect, and *d* ≥ 0.8 a large effect.

Secondary analyses examined potential moderating effects of demographic variables through independent samples *t*-tests. For the biological age and years spent driving professionally analyses, the sample population was divided into two groups to enable comparison between groups. This division was achieved by finding the median value, which was approximately 22.5 years for biological age and 7.5 years for professional racing experience. This median split approach, while subject to criticism for information loss and reduced power (MacCallum et al., 2002) was selected for the secondary analyses given the exploratory nature of these comparisons and the need to maintain interpretive consistency with the primary F1 versus F2 categorical comparison. The small sample size precluded more sophisticated continuous analyses that would require adequate statistical power. As a supplementary sensitivity analysis, Pearson correlations between years of professional experience (treated as a continuous variable) and each ACSI-28 subscale were computed; these exploratory correlations should be interpreted cautiously given the limited sample size and are presented in supplementary materials. Age-based comparisons therefore employed drivers above the median age (>22.5 years) versus those below the median age (≤22.5 years), while experience-based analyses utilized the 7.5-year cutoff to distinguish between more experienced (>7.5 years) and less experienced (≤7.5 years) drivers. Given the exploratory nature of the study and small sample size, interpretation focused on effect size magnitude alongside statistical significance, with acknowledgment of potential Type I error inflation due to multiple comparisons across seven psychological factors. While additional data would be required to increase the robustness of the results and enable analysis with more than two groups, the current approach is statistically sound and based on existing literature, providing a reliable indication of likely outcomes with expanded sample sizes.

Post-hoc power analysis using G*Power 3.1 (Faul et al., 2007) revealed that with *n* = 5 per group (*α* = 0.05, two-tailed), the study achieved statistical power of approximately 17% for detecting medium effects (*d* = 0.50), 31% for detecting medium-large effects (*d* = 0.65), and 52% for detecting large effects (*d* = 0.80). Adequate power (≥80%) was achieved only for very large effect sizes (*d* ≥ 1.20). Consequently, non-significant findings for coping with adversity (*d* = 0.63) and peaking under pressure (*d* = 0.56) should not be interpreted as evidence of null effects; rather, these medium effect sizes may represent meaningful differences that failed to reach significance due to insufficient statistical power. Interpretation therefore emphasizes effect size magnitude alongside statistical significance, recognizing that absence of significance does not equate to absence of effect in underpowered designs.

## Results

### Descriptive statistics

**Figure 1:**
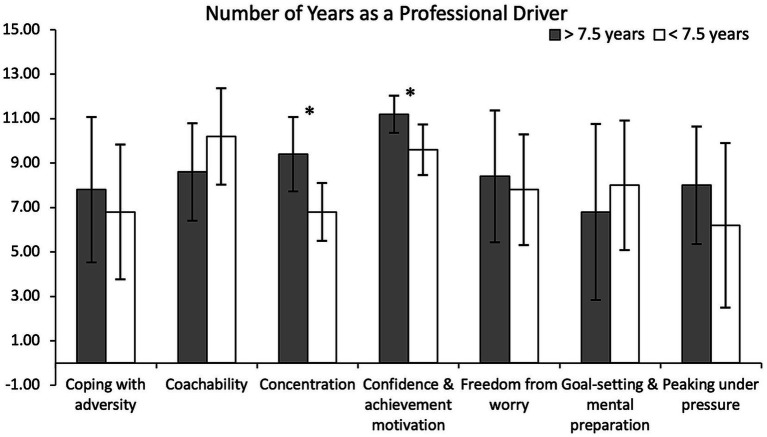
Bar chart displaying mean scores (±SD) for the seven ACSI-28 psychological skill subscales, comparing Formula 1 and Formula 2 drivers. *Statistically significant difference between groups (*p* < 0.05).

Table 2 presents descriptive statistics for all seven ACSI-28 psychological factors by racing series (see Figure 1). F1 drivers demonstrated higher mean scores than F2 drivers across five of the seven psychological factors, with F2 drivers showing advantages only in Goal Setting and Mental Preparation and Freedom from Worry. The total ACSI score revealed F1 drivers scored higher overall (M = 60.2, SD = 8.61) compared to F2 drivers (M = 55.4, SD = 11.19), though this difference was not statistically significant [*t*(8) = 0.76, *p* = 0.47].

**Table 2:** Descriptive statistics and independent *t*-test results comparing ACSI-28 psychological skill subscales between Formula 1 and Formula 2 drivers.

F1 vs F2								
Variable	Racing Series	*N*	Mean	SD	*t*-value	df	*p*-value	Cohen’s *d*
Coping with adversity	F1	5	7.80	3.27	0.50	8.00	0.63	0.63
	F2	5	6.80	3.03				
Coachability	F1	5	8.60	2.19	−1.16	8.00	0.28	0.73
	F2	5	10.20	2.17				
Concentration	F1	5	9.40	1.67	2.74	8.00	0.03^*^	1.31^*^
	F2	5	6.80	1.30				
Confidence and achievement motivation	F1	5	11.20	0.84	2.53	8.00	0.04^*^	1.60^*^
	F2	5	9.60	1.14				
Freedom from worry	F1	5	8.40	2.97	0.35	8.00	0.74	0.22
	F2	5	7.80	2.49				
Goal-setting and mental preparation	F1	5	6.80	3.96	−0.55	8.00	0.60	0.34
	F2	5	8.00	2.92				
Peaking under pressure	F1	5	8.00	2.65	0.88	8.00	0.40	0.56
	F2	5	6.20	3.70				
Total score	F1	5	60.20	8.61	0.76	8.00	0.47	0.48
	F2	5	55.40	11.19				

* Statistically significant (*p* < 0.05); Cohen’s *d* interpretation: 0.2–0.5 = small, 0.5–0.8 = medium, ≥0.8 = large effect.

### Primary analysis: F1 versus F2 comparisons

#### Statistically significant differences

Two psychological factors demonstrated statistically significant differences between F1 and F2 drivers. Concentration showed the most pronounced difference, with F1 drivers scoring significantly higher (M = 9.4, SD = 1.67) than F2 drivers (M = 6.8, SD = 1.30), *t*(8) = 2.74, *p* = 0.03, representing a large effect size (*d* = 1.31). This finding indicates F1 drivers possess substantially superior attentional control and ability to focus under racing conditions.

Confidence and Achievement Motivation also revealed a significant advantage for F1 drivers (M = 11.2, SD = 0.84) compared to F2 drivers (M = 9.6, SD = 1.14), *t*(8) = 2.53, *p* = 0.04, with the largest effect size observed in the study (*d* = 1.60). This suggests F1 drivers demonstrate markedly higher confidence in their ability and intrinsic motivation compared to their F2 counterparts.

#### Non-significant differences with notable effect sizes

Several psychological factors showed meaningful differences despite lacking statistical significance. Coping with Adversity favoured F1 drivers (M = 7.8, SD = 3.27) over F2 drivers (M = 6.8, SD = 3.03), *t*(8) = 0.50, *p* = 0.63, with a medium effect size (*d* = 0.63). Similarly, Peaking Under Pressure showed F1 drivers (M = 8.0, SD = 2.65) outperforming F2 drivers (M = 6.2, SD = 3.70), *t*(8) = 0.88, *p* = 0.40, also with a medium effect size (*d* = 0.56).

#### Areas of F2 advantage

F2 drivers demonstrated advantages in two psychological areas. Goal Setting and Mental Preparation showed F2 drivers scoring higher (M = 8.0, SD = 2.92) than F1 drivers (M = 6.8, SD = 3.96), *t*(8) = −0.55, *p* = 0.60, with a small effect size (*d* = 0.34). Freedom from Worry also favoured F2 drivers (M = 7.8, SD = 2.49) compared to F1 drivers (M = 8.4, SD = 2.97), *t*(8) = 0.35, *p* = 0.74, though with a small effect size (*d* = 0.22).

#### No group differences

Coachability showed virtually identical scores between groups, with both F1 (M = 8.6, SD = 2.19) and F2 drivers (M = 10.2, SD = 2.17) demonstrating similar receptiveness to feedback and instruction, *t*(8) = −1.16, *p* = 0.28, resulting in a negligible effect size (*d* = 0.00).

### Secondary analyses: demographic moderators

#### Age-based comparisons

**Figure 2:**
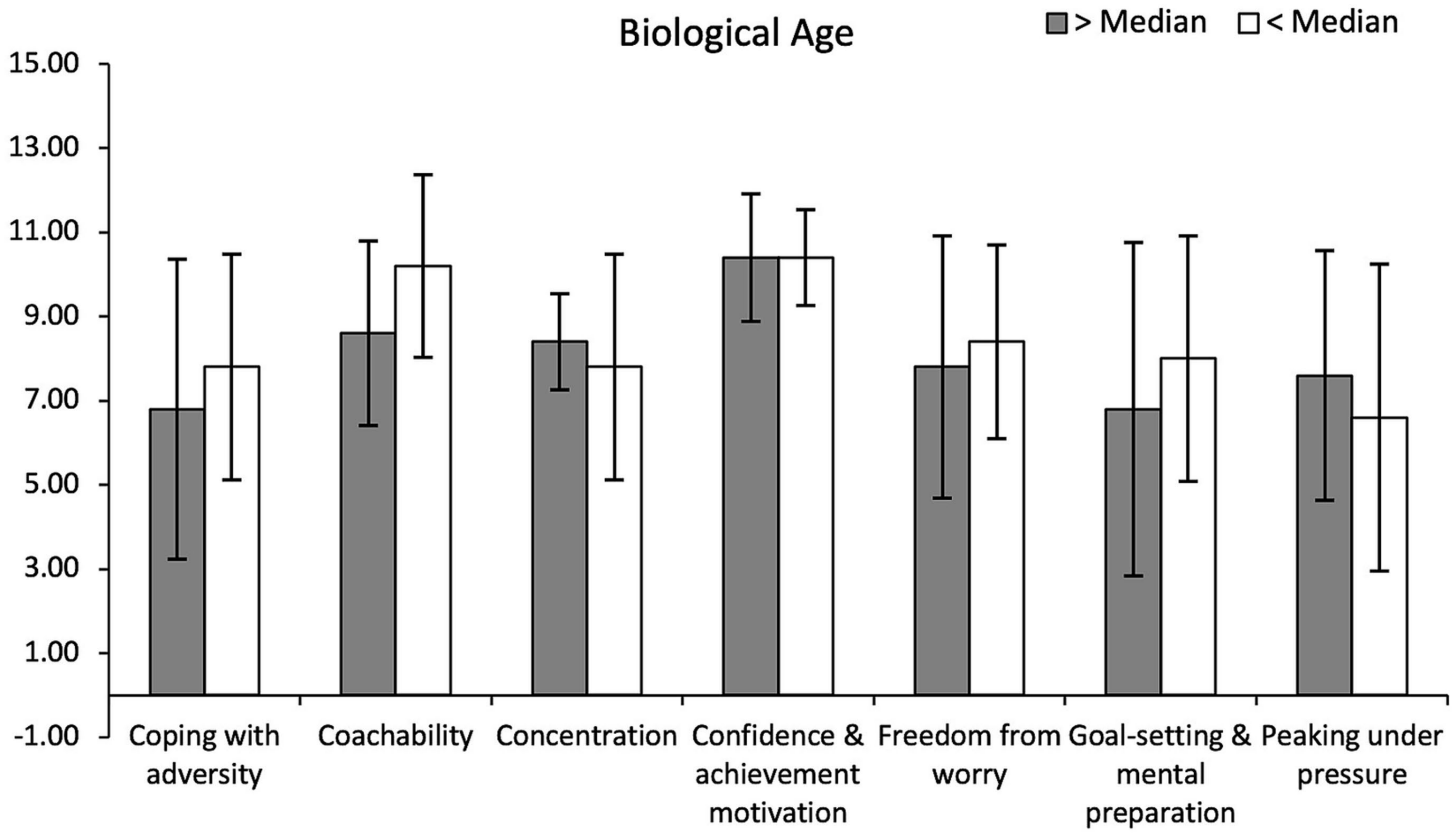
Bar chart displaying mean scores (±SD) for the seven ACSI-28 psychological skill subscales, comparing drivers above and below the median age.

Age-based analyses using median split methodology revealed no statistically significant differences across any psychological factors (see Table 3 and Figure 2). Drivers above the median age showed similar psychological profiles to those below the median across all seven factors, with the largest difference observed in Concentration (above median: M = 8.4, SD = 1.14; below median: M = 7.8, SD = 2.68), *t*(8) = 0.46, *p* = 0.66. Total ACSI scores were comparable between age groups (above median: M = 56.4, SD = 10.50; below median: M = 59.2, SD = 9.93), *t*(8) = −0.43, *p* = 0.68.

**Table 3:** Descriptive statistics and independent *t*-test results comparing ACSI-28 psychological skill subscales between drivers above and below the median age.

Age								
Variable	Age	*N*	Mean	SD	*t*-value	df	*p*-value	Cohen’s *d*
Coping with adversity	>Median	5	6.80	3.56	−0.50	8.00	0.63	0.32
	<Median	5	7.80	2.68				
Coachability	>Median	5	8.60	2.19	−1.16	8.00	0.28	0.73
	<Median	5	10.20	2.17				
Concentration	>Median	5	8.40	1.14	0.46	8.00	0.66	0.29
	<Median	5	7.80	2.68				
Confidence and achievement motivation	>Median	5	10.40	1.52	0.00	8.00	1.00	0.00
	<Median	5	10.40	1.14				
Freedom from worry	>Median	5	7.80	3.11	−0.34	8.00	0.74	0.22
	<Median	5	8.40	2.30				
Goal-setting and mental preparation	>Median	5	6.80	3.96	−0.55	8.00	0.60	0.34
	<Median	5	8.00	2.92				
Peaking under pressure	>Median	5	7.60	2.97	0.48	8.00	0.65	0.30
	<Median	5	6.60	3.65				
Total score	>Median	5	56.40	10.50	−0.43	8.00	0.68	0.27
	<Median	5	59.20	9.93				

No statistically significant differences were observed. Cohen’s *d* interpretation: 0.2–0.5 = small, 0.5–0.8 = medium, ≥0.8 = large effect.

#### Experience-based comparisons

**Figure 3:**
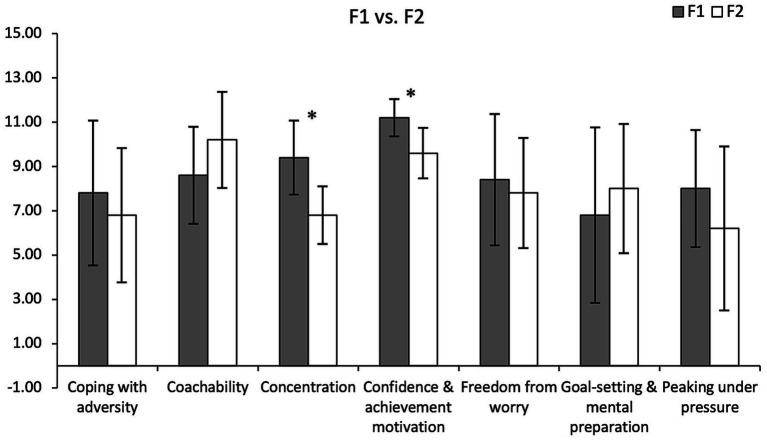
Bar chart displaying mean scores (±SD) for the seven ACSI-28 psychological skill subscales, comparing drivers with more than 7.5 years and less than 7.5 years of professional driving experience. *Statistically significant difference between groups (*p* < 0.05).

Years of professional driving experience (using 7.5-year cutoff) revealed patterns similar to the primary F1 versus F2 analysis (see Table 4 and Figure 3). Drivers with greater than 7.5 years of experience demonstrated significantly higher Concentration scores (M = 9.4, SD = 1.67) compared to less experienced drivers (M = 6.8, SD = 1.30), *t*(8) = 2.74, *p* = 0.03. Additionally, more experienced drivers showed significantly higher Confidence and Achievement Motivation (M = 11.2, SD = 0.84) than less experienced drivers (M = 9.6, SD = 1.14), *t*(8) = 2.53, *p* = 0.04. These findings mirror the F1 versus F2 differences, suggesting that experience and racing series level may be confounded variables, with F1 drivers generally having more professional experience than F2 drivers.

**Table 4:** Descriptive statistics and independent *t*-test results comparing ACSI-28 psychological skill subscales between drivers with more and less than 7.5 years of professional driving experience.

Number of years of professional driving								
Variable	Experience	*N*	Mean	SD	*t*-value	df	*p*-value	Cohen’s *d*
Coping with adversity	>7.5 years	5	7.80	3.27	0.50	8.00	0.63	0.63
	<7.5 years	5	6.80	3.03				
Coachability	>7.5 years	5	8.60	2.19	−1.16	8.00	0.28	0.73
	<7.5 years	5	10.20	2.17				
Concentration	>7.5 years	5	9.40	1.67	2.74	8.00	0.03^*^	1.31^*^
	<7.5 years	5	6.80	1.30				
Confidence and achievement motivation	>7.5 years	5	11.20	0.84	2.53	8.00	0.04^*^	1.60^*^
	<7.5 years	5	9.60	1.14				
Freedom from worry	>7.5 years	5	8.40	2.97	0.35	8.00	0.73	0.22
	<7.5 years	5	7.80	2.49				
Goal-setting and mental preparation	>7.5 years	5	6.80	3.96	−0.55	8.00	0.60	0.34
	<7.5 years	5	8.00	2.92				
Peaking under pressure	>7.5 years	5	8.00	2.65	0.88	8.00	0.40	0.56
	<7.5 years	5	6.20	3.70				
Total score	>7.5 years	5	60.20	8.61	0.76	8.00	0.47	0.48
	<7.5 years	5	55.40	11.19				

* Statistically significant (*p* < 0.05); Cohen’s *d* interpretation: 0.2–0.5 = small, 0.5–0.8 = medium, ≥0.8 = large effect.

## Discussion

### Summary of key findings

This exploratory pilot study represents the first published examination of psychological skills in Formula 1 and Formula 2 drivers, addressing a critical gap in motorsport psychology research. While the small sample size (*n* = 10) necessitates cautious interpretation, the study’s access to 25% of each championship’s driver roster provides unique preliminary insights that establish empirical foundations for future large-scale investigations. The ACSI-28 assessment revealed a nuanced psychological profile distinguishing these elite motorsport competitors, with several key findings emerging that have both theoretical and practical implications for understanding elite performance under extreme pressure conditions.

While no statistically significant overall difference was observed between F1 and F2 drivers on the total ACSI-28 score (*p* = 0.47), meaningful distinctions emerged across specific psychological skill domains. F1 drivers demonstrated statistically significant superiority in two critical areas: Concentration (M = 9.4 vs. 6.8, *d* = 1.31, *p* = 0.03) and Confidence and Achievement Motivation (M = 11.2 vs. 9.6, *d* = 1.60, *p* = 0.04). These findings suggest that while both driver groups possess comparable overall psychological coping resources, F1 drivers exhibit marked advantages in the specific mental skills most crucial for elite performance under extreme pressure, consistent with theoretical frameworks emphasizing psychological skills as fundamental determinants of performance, consistency, and resilience rather than merely supportive tools (Vealey, 2007).

The effect size analysis revealed a clear hierarchy of psychological factors beyond the statistically significant domains. F1 drivers showed medium effect advantages in Coping with Adversity (*d* = 0.63) and Peaking Under Pressure (*d* = 0.56), despite non-significant *p*-values likely due to the study’s limited statistical power. Conversely, F2 drivers demonstrated advantages in Goal Setting and Mental Preparation (*d* = 0.34) and Freedom from Worry (*d* = 0.22), while Coachability showed negligible differences between groups (*d* = 0.00). The secondary analyses revealed that biological age showed no relationship to psychological skills, while years of professional racing experience mirrored the F1 versus F2 pattern, suggesting that competitive level and experience may be confounded variables in this elite population.

### Interpretation and cross-sport literature comparison

The observed superiority of F1 drivers in Concentration aligns strongly with established sport psychology literature emphasizing attentional control as a fundamental determinant of elite performance. The large effect size (*d* = 1.31) suggests that F1 drivers possess attentional capabilities that extend substantially beyond general athletic populations. This finding resonates particularly well with (Young and Knight, 2014) study of risk sport athletes, which found that experienced performers scored significantly higher than novice athletes on concentration, coping with adversity, and freedom from worry subscales. The parallel between motorsport and risk sports is theoretically meaningful, as both involve unpredictable and dangerous sporting environments where psychological skills serve as precautionary behaviour for managing extreme sporting conditions (Young and Knight, 2014). The extreme consequences of attentional lapses in motorsport, where split-second decisions at speeds exceeding 350 km/h can determine both performance outcomes and safety considerations, may create selective pressures that favour drivers with superior concentration abilities. Research with elite volleyball players similarly revealed that elite performers recorded higher scores across all mental skill dimensions, demonstrating that psychological skills play increasingly important roles in reaching peak performance levels as competitive demands intensify (Mohammadzadeh and Sami, 2014).

The substantial advantage shown by F1 drivers in Confidence and Achievement Motivation (*d* = 1.60, the largest effect size in the study) provides strong support for theoretical frameworks emphasizing self-efficacy as a cornerstone of elite performance. This finding aligns with broader sport psychology literature showing that more successful athletes identified themselves as less depressed, less sensitive, yet more confident, more anxious and aggressive than the less successful athletes across various sports contexts (Bebetsos, 2003; Karamousalidis et al., 2006; Mahoney, 1989). The magnitude of the confidence difference suggests that reaching motorsport’s pinnacle requires not merely technical competence, but unwavering belief in one’s capabilities under extreme performance pressures. Bebetsos (2015) study of elite archery athletes provides particularly relevant cross-sport validation, as the finding that more experienced competitors showed increased concentration and confidence indicators directly parallels the current results.

The concentration superiority demonstrated by F1 drivers parallels psychological demands across other life-critical occupations. Military pilots operating in single-seat multirole fighter aircraft are exposed to a demanding cognitive work environment, implying intense physical and psychological stress and fatigue (Rosa et al., 2022), while neuroimaging research on F16 fighter pilots reveals that findings of increased global connectivity in frontal regions in more experienced pilots might reflect the cognitive demands to overcome the challenges of operating a fighter jet (Radstake et al., 2023) Similarly, air traffic controllers managing aircraft safety demonstrate that air traffic controllers simultaneously develop complex and multiple tasks in the course of their activities, with concern raised over the high level of attention needed by these professionals (Metzger and Parasuraman, 2006), and empirical studies confirm that mental workload affects time perception in real aviation scenarios (Truschzinski et al., 2018). Research consistently indicates that impaired attention, slower reaction time, reduced vigilance and problem-solving directly impact safety across aviation contexts (Grassmann et al., 2017), suggesting that the concentration advantages distinguishing F1 drivers represent fundamental cognitive adaptations necessary across high-stakes domains where split-second decisions determine both performance outcomes and safety consequences.

An important methodological consideration emerged regarding the seemingly contradictory findings for Coachability and Confidence and Achievement Motivation, both of which showed identical 1.6-point mean differences between groups. While Coachability demonstrated high within-group variability (F1: SD = 2.19; F2: SD = 2.17), Confidence showed markedly low variability (F1: SD = 0.84; F2: SD = 1.14). This difference in variability patterns explains why identical mean differences yielded vastly different statistical outcomes (Coachability: *d* = 0.00, *p* = 0.28; Confidence: *d* = 1.60, *p* = 0.04). The high variability in coachability suggests this skill may be influenced more by individual differences than competitive level, while the low variability in confidence indicates this construct is closely tied to racing series demands (F1 vs. F2). These patterns underscore the importance of considering both effect sizes and variability when interpreting psychological skill differences in elite populations.

The medium effect sizes observed for Coping with Adversity (*d* = 0.63) and Peaking Under Pressure (*d* = 0.56), though not statistically significant, align with Lazarus and Folkman’s foundational coping framework and merit theoretical consideration. Elite motorsport epitomises pressure environments where drivers may be confronted with situations, where the outcome hinges on one pressured moment and must maintain split-second decisions, maintain fine motor control under physical and mental fatigue—underpinned by the knowledge that the performance outcome can result in consequences of risk or reward (Kent et al., 2018). The non-significant findings may reflect the study’s limited statistical power rather than absence of meaningful differences, particularly given that (Young and Knight, 2014) found significant differences in coping with adversity between experienced and novice risk sport athletes, suggesting that larger sample sizes might reveal stronger evidence for F1 drivers’ superior pressure-coping capabilities.

### Developmental progression and F2 advantages

The intriguing finding that F2 drivers showed advantages in Goal Setting and Mental Preparation can be interpreted through established theoretical frameworks of expertise development in sport (Lange-Smith et al., 2024; Reinebo et al., 2024). Côté and colleagues’ Developmental Model of Sport Participation (DMSP; Côté, 1999; Côté et al., 2007) proposes that athletes progress through distinct developmental stages characterized by different psychological emphases. The current findings align with this framework: F2 drivers, positioned in what might be conceptualized as the ‘investment years’ of their motorsport career trajectory, demonstrate systematic preparation behaviors consistent with deliberate practice orientations. In contrast, F1 drivers, having achieved elite status, may have transitioned to a ‘maintenance’ phase characterized by automatic execution and pressure-response optimization rather than explicit preparatory activities. This interpretation is further supported by Ericsson’s (2006) deliberate practice framework and Collins and MacNamara’s (2017) talent development model, both of which suggest that the psychological skills required for skill acquisition differ qualitatively from those required for expert performance maintenance.

The observed advantage of F2 drivers in Goal Setting and Mental Preparation may be partially explained by the structural differences in competitive scheduling between the two series. F1 drivers operate within a compressed, intensive racing calendar that creates a maintenance-focused approach throughout the season, requiring them to sustain peak psychological and physical readiness while transitioning rapidly from race to race with minimal recovery periods. This demanding schedule necessitates a ‘holding pattern’ mentality where drivers prioritize consistency, freshness, and immediate performance readiness over extensive goal-setting and systematic preparation activities. Conversely, F2’s reduced race frequency creates substantial inter-race intervals of 4–5 weeks, providing meaningful opportunities for comprehensive training blocks, detailed performance analysis, and systematic goal-setting processes. These extended gaps allow F2 drivers to engage in the deliberate, structured preparation activities that may become less feasible at F1’s relentless competitive pace, potentially explaining the systematic preparation advantages observed in the current study.

The F2 advantage in systematic preparation, coupled with F1 advantages in pressure response and concentration, suggests a developmental progression in psychological skill emphasis rather than simple superiority across all domains, with important implications for talent development programs suggesting that maintaining systematic preparation skills while developing pressure-response capabilities and attentional control may represent an optimal developmental pathway.

The negligible difference in Coachability between groups (*d* = 0.00) provides important theoretical insights into the universal nature of certain psychological skills, suggesting that receptiveness to feedback and instruction remains consistently important across both competitive levels and supporting theoretical frameworks emphasizing the ongoing nature of skill development even at elite levels. This result aligns with research demonstrating that psychological skills represent trainable constructs that can be systematically developed through appropriate intervention programs, rather than fixed traits that determine competitive level. The universal importance of coachability across F1 and F2 drivers supports the implementation of comprehensive psychological support programs throughout the developmental pathway, rather than restricting such services to elite levels.

### Evidence-based psychological skills training implications

The current findings should be interpreted within the context of strong meta-analytic evidence supporting psychological skills training (PST) interventions. Lochbaum et al.’s (2022) comprehensive meta-analyses covering 16 distinct sport psychology constructs demonstrated that sport psychology interventions enhanced performance outcomes, while Lange-Smith et al. (2024) review concluded that PST interventions can enhance performance, and (Reinebo et al., 2024) systematic review found significant moderate effects for psychological skills training, mindfulness-based approaches, and imagery techniques in competitive athlete performance. This evidence base provides strong theoretical and empirical support for targeted psychological interventions in motorsport contexts, with the specific pattern of F1 advantages in concentration and confidence suggesting that interventions targeting these domains may be particularly effective for F2 drivers aspiring to F1 progression.

Bochaver et al. (2021) found that only five out of twelve mental skills showed significant development as athletes progressed from novice to experienced levels, while seven skills did not improve automatically with experience has particular relevance for motorsport psychology. This research demonstrates that psychological skills require deliberate training interventions rather than developing naturally through experience alone, supporting the need for systematic PST programs rather than relying solely on competitive experience for psychological development. The current study’s finding that F2 drivers excel in systematic preparation while F1 drivers show superior pressure response and concentration suggests that different psychological skills may require different developmental approaches, with systematic preparation skills benefiting from explicit training and education, while pressure-response capabilities may require exposure-based interventions and high-pressure simulation training.

### Study limitations and methodological considerations

While the sample size of 10 participants (5 per group) may appear limited in absolute terms, it represents a substantial proportion of the elite motorsport population, comprising 25% of Formula 1 drivers (5 out of 20 drivers) and approximately 23% of Formula 2 drivers (5 out of 22 drivers) competing in the 2019 season. Access to elite motorsport athletes presents unique methodological challenges that significantly constrain sample sizes, as Formula 1 and Formula 2 represent the absolute pinnacle of single-seater motorsport with severely limited participant pools. The demanding schedules, global travel commitments, commercial obligations, and protective team environments create substantial barriers to research participation. Young and Knight (2014) acknowledged similar challenges in their risk sport research, noting that a sample of over 200 athletes is noteworthy while simultaneously recognizing that a larger sampling of risk sport athletes would help to solidify the extent to which psychological skills are employed. The current study’s achievement in securing participation from 25% of each championship’s driver roster represents a significant methodological accomplishment given these access constraints.

The limited statistical power resulting from small sample sizes remains a legitimate concern, particularly for detecting medium effect sizes. However, the study was adequately powered for detecting the large effects observed in concentration and confidence domains, and the substantial effect sizes for pressure-related skills (Coping with Adversity, *d* = 0.63; Peaking Under Pressure, *d* = 0.56) suggest meaningful differences that would likely achieve significance with larger samples. The small sample size further amplifies the importance of examining variability patterns and effect sizes alongside statistical significance, as demonstrated by the contrasting outcomes for Coachability and Confidence despite identical mean differences. The cross-sectional design prevents causal inferences about the relationship between psychological skills and competitive level, as the data cannot distinguish between inherent psychological characteristics that enabled F1 progression versus skills developed as a consequence of competing at the highest level. Longitudinal research tracking drivers throughout their career progression would provide stronger evidence for causal relationships and illuminate the developmental trajectories of psychological skills in motorsport contexts. A notable methodological consideration that influenced statistical outcomes concerns the differential variability patterns across psychological constructs. The seemingly contradictory findings for Coachability and Confidence and Achievement Motivation, both of which showed identical 1.6-point mean differences between groups, illustrate this point. While Coachability demonstrated high within-group variability (F1: SD = 2.19; F2: SD = 2.17), Confidence showed markedly low variability (F1: SD = 0.84; F2: SD = 1.14). This difference in variability patterns explains why identical mean differences yielded vastly different statistical outcomes (Coachability: *d* = 0.73, *p* = 0.28; Confidence: *d* = 1.60, *p* = 0.04). The high variability in coachability suggests this skill may be influenced more by individual differences than competitive level, while the low variability in confidence indicates this construct is closely tied to racing series demands. These patterns underscore the importance of considering both effect sizes and variability when interpreting psychological skill differences in elite populations.

The reliance on self-report measures through the ACSI-28, while psychometrically sound, introduces potential social desirability bias and may not capture the full spectrum of psychological skills relevant to motorsport performance, as it does not assess certain skills potentially crucial for motorsport, such as specific risk assessment capabilities, spatial processing under stress, or motorsport-specific anxiety management techniques. Timing of data collection during the competitive season may have influenced psychological state and self-reporting accuracy, as drivers’ responses could be affected by recent performance outcomes or current competitive standings. The demographic limitations, including the absence of female drivers and narrow age range, reflect current motorsport demographics but limit generalizability to broader populations, highlighting the need for gender-inclusive research as female participation in elite motorsport continues to evolve.

### Future research directions

Longitudinal studies tracking drivers from junior categories through their professional careers represent the most critical future research direction, illuminating the developmental trajectory of psychological skills, identifying critical transition points, and determining whether psychological advantages precede competitive success or develop through elite-level experience. Multi-wave longitudinal designs could examine how psychological skills change during career transitions (e.g., junior formulae to F2, F2 to F1) and identify predictive factors for successful progression, providing definitive evidence for causal relationships and immediate practical applications for talent identification and development programs.

Randomized controlled trials examining the effectiveness of targeted psychological skills training interventions represent crucial next steps for the field. Given the current study’s findings, interventions targeting concentration enhancement and confidence building appear particularly promising for F2 drivers aspiring to F1 progression. The integration of technology-enhanced training methods, including virtual reality simulation and biofeedback systems, offers particular promise for motorsport applications given the sport’s technological sophistication. Systematic evaluation of existing psychological support programs within motorsport organizations could provide valuable insights into effective intervention strategies and inform evidence-based practice guidelines.

Comparative studies examining psychological skills across different motorsport disciplines (Formula racing, endurance racing, rally, etc.) would illuminate the sport-specific versus general nature of elite psychological capabilities and inform the development of sport-specific psychological assessment and intervention protocols. Comparisons with other high-risk, high-precision sports could provide broader theoretical insights into psychological skills in extreme performance environments, building on (Young and Knight, 2014) finding that risk sport athletes utilize psychological skills “as a form of precautionary behaviour, which prepare risk sport athletes for future adversity” (p. 7).

The exploration of psychological factors influencing safety behaviours and risk management represents a theoretically and practically important research direction unique to motorsport. Research examining the relationship between psychological skills and objective safety metrics (accident involvement, safety protocol compliance, risk-taking behaviours) could provide evidence for the dual performance-safety benefits of psychological skills training. As female participation in elite motorsport continues to evolve, gender-focused research examining psychological skills in female competitors will become increasingly important, while research across diverse cultural and socioeconomic backgrounds could inform more inclusive talent identification and development programs.

### Practical applications and industry implications

The practical implications of this research extend beyond performance optimization to encompass critical safety considerations unique to motorsport. Unlike traditional sporting contexts where psychological interventions primarily target competitive outcomes, concentration training and confidence-building interventions in motorsport serve a dual function: enhancing competitive performance while simultaneously strengthening the cognitive vigilance systems that prevent potentially catastrophic errors. This dual performance-safety framework should inform the development, implementation, and evaluation of psychological skills training programs in motorsport contexts, with intervention efficacy measured not only through performance metrics but also through safety-relevant outcomes such as incident frequency, error rates, and risk management behaviors.

The findings carry significant implications for evidence-based driver development and selection processes within motorsport organizations. The substantial advantages shown by F1 drivers in concentration and confidence suggest these domains should receive priority attention in development programs targeting F1 progression, with psychological assessment protocols emphasizing these abilities to enhance driver selection accuracy and identify specific developmental priorities.

For F2 drivers aspiring to F1 progression, the results suggest maintaining strengths in systematic preparation while implementing targeted interventions for concentration enhancement and confidence building. Training programs could leverage F2 drivers’ goal-setting advantages as a foundation for developing other psychological skills, creating developmentally appropriate progression pathways. The equal coachability across groups supports implementing comprehensive psychological skills training programs at both competitive levels, suggesting that psychological interventions would be equally well-received throughout the developmental pathway.

The study establishes an empirical foundation for evidence-based psychological support in motorsport, addressing the significant research gap identified in the literature. The specific psychological skill profiles could inform standardized psychological assessment protocols for motorsport contexts and guide resource allocation in driver development initiatives. Concentration training programs, confidence-building interventions, and pressure simulation training emerge as priority areas for psychological support services based on the current findings and broader PST literature.

Early identification programs could incorporate concentration and confidence assessments alongside traditional performance metrics, potentially improving talent identification accuracy. Development programs could be structured to address the specific psychological skill patterns observed at different competitive levels, with systematic preparation emphasized in junior categories and concentration/confidence development prioritized as drivers progress toward elite levels.

The dual performance-safety implications of psychological skills in motorsport create unique opportunities for integrated intervention approaches. The concentration and confidence advantages observed in F1 drivers may reflect not only performance optimization but also safety-related competencies, suggesting that psychological skills training could contribute to both competitive success and safety culture development within motorsport organizations, providing compelling value propositions for both competitive and regulatory stakeholders.

## Conclusion

This exploratory pilot study provides preliminary evidence regarding psychological skill differences between Formula 1 and Formula 2 drivers. Two statistically significant findings emerged: F1 drivers demonstrated superior Concentration (*d* = 1.31, *p* = 0.03) and Confidence and Achievement Motivation (*d* = 1.60, *p* = 0.04). Medium effect sizes favoring F1 drivers in Coping with Adversity (*d* = 0.63) and Peaking Under Pressure (*d* = 0.56) warrant investigation in adequately powered studies. These preliminary findings, while requiring replication with larger samples, suggest that specific psychological capabilities may distinguish the highest tier of motorsport performance.

The study’s identification of F2 drivers’ strengths in goal-setting and mental preparation highlights the systematic, developmental nature of this feeder series, suggesting a progression model where systematic preparation capabilities provide foundations for developing pressure-response skills and attentional control. The universal importance of coachability across both levels underscores the ongoing nature of elite athlete development and supports comprehensive psychological support throughout the developmental pathway. Importantly, the absence of age-related differences suggests that psychological skill development, rather than mere chronological maturation or experience accumulation, drives mental performance advancement in elite motorsport.

The theoretical integration of performance and safety implications represents a unique contribution to sport psychology literature, as motorsport’s extreme environment creates contexts where mental readiness influences both competitive outcomes and safety considerations simultaneously. From a practical perspective, this research indicates that mental training should receive equal emphasis alongside technical and physical preparation for aspiring Formula 1 drivers, opening new avenues for targeted psychological interventions, talent identification protocols, and safety-focused applications.

The study’s methodological achievement in securing participation from 25% of both championship rosters demonstrates the feasibility of elite motorsport psychology research despite significant access challenges. Future research expanding these findings with larger samples, longitudinal designs, and intervention studies will further illuminate the developmental pathways and causal relationships underlying elite motorsport psychological performance. Ultimately, this research contributes essential knowledge to an understudied but critically important domain of elite athletic performance, establishing empirical foundations for evidence-based psychological support and demonstrating that reaching Formula 1 level requires not merely technical and physical excellence, but specific psychological capabilities that enable consistent performance under the most extreme competitive pressures.

## Data Availability

The original contributions presented in the study are included in the article/supplementary material, further inquiries can be directed to the corresponding author.
